# Tracing dsDNA Virus–Host Coevolution through Correlation of Their G-Quadruplex-Forming Sequences

**DOI:** 10.3390/ijms22073433

**Published:** 2021-03-26

**Authors:** Natália Bohálová, Alessio Cantara, Martin Bartas, Patrik Kaura, Jiří Šťastný, Petr Pečinka, Miroslav Fojta, Václav Brázda

**Affiliations:** 1 Institute of Biophysics of the Czech Academy of Sciences, Královopolská 135, 612 65 Brno, Czech Republic; natalia.bohalova@ibp.cz (N.B.); alexcantara41@gmail.com (A.C.); fojta@ibp.cz (M.F.); 2 Department of Experimental Biology, Faculty of Science, Masaryk University, Kamenice 5, 625 00 Brno, Czech Republic; 3 Department of Biology and Ecology, Institute of Environmental Technologies, Faculty of Science, University of Ostrava, 710 00 Ostrava, Czech Republic; martin.bartas@osu.cz (M.B.); petr.pecinka@osu.cz (P.P.); 4 Faculty of Mechanical Engineering, Brno University of Technology, Technická 2896/2, 616 69 Brno, Czech Republic; 160702@vutbr.cz (P.K.); stastny@fme.vutbr.cz (J.Š.); 5 Department of Informatics, Mendel University in Brno, Zemědělská 1, 613 00 Brno, Czech Republic

**Keywords:** G-quadruplex, virus, bioinformatics, coevolution, host, dsDNA, G4Hunter

## Abstract

The importance of gene expression regulation in viruses based upon G-quadruplex may point to its potential utilization in therapeutic targeting. Here, we present analyses as to the occurrence of putative G-quadruplex-forming sequences (PQS) in all reference viral dsDNA genomes and evaluate their dependence on PQS occurrence in host organisms using the G4Hunter tool. PQS frequencies differ across host taxa without regard to GC content. The overlay of PQS with annotated regions reveals the localization of PQS in specific regions. While abundance in some, such as repeat regions, is shared by all groups, others are unique. There is abundance within introns of Eukaryota-infecting viruses, but depletion of PQS in introns of bacteria-infecting viruses. We reveal a significant positive correlation between PQS frequencies in dsDNA viruses and corresponding hosts from archaea, bacteria, and eukaryotes. A strong relationship between PQS in a virus and its host indicates their close coevolution and evolutionarily reciprocal mimicking of genome organization.

## 1. Introduction

Viruses are intracellular parasites closely coevolving with their host organisms and thus shaping genotypic diversity [1,2]. The interplay between a virus and its host constitutes a powerful mechanism of reciprocal selection pressure. Coevolution of the two can be traced by nucleic acid sequence, protein tertiary structure, and also at the whole-function level. For example, hosts’ antiviral defense mechanisms often originate from viruses [2,3,4]. The study of reciprocal coevolutionary adaptations between a virus and the host immune system could provide new insights and potential strategies in developing antiviral treatments [5].

G-quadruplexes (G4s) are noncanonical, local secondary structures of nucleic acids that have been identified as having regulatory roles within cells in gene expression, replication, and telomere maintenance [6,7,8]. A G4 consists of stacked planar G-quartets, which are built by Hoogsteen hydrogen bond-based pairing of four guanines. It has been demonstrated that G4s are very often targets for various cellular proteins [9,10,11], and a specific domain for G4 recognition has been shown [12,13]. Moreover, several proteins are also capable of stabilizing the G4 structure [14,15]. Recently, it has been demonstrated that the G4 binding domain is also conserved between SARS-CoV and SARS-CoV-2 genomes [16], and it was proven to be crucial for the SARS-CoV life cycle [17]. G4s can be found in all domains of life [18,19,20,21], and they have been described as constituting an important structural genomic feature with various functions in several viral classes [21,22]. The G4 formation was shown to limit the replication and transcription of the Ebola virus, hepatitis B virus (HBV), hepatitis C virus (HCV), human immunodeficiency virus (HIV), and several viruses from the *Herpesviridae* family [23,24,25,26]. In the life cycles of the Epstein–Barr virus (EBV) and Kaposi sarcoma herpesvirus (KSHV), moreover, RNA G4 has been described as a cis-acting regulatory element that downregulates the translation of highly antigenic proteins and thus influences the immune evasion of the virus and eases transit and persistence in the latent period of infection [27,28]. Importantly, the functions of G4 may be modified via their stabilization by proteins or small-molecule ligands [29,30]. Therefore, stabilizing G4 ligands are considered promising antiviral and antibacterial drugs [29]. Coevolution of viral and host loop sequences of the G-quadruplex-forming sequences in human *Herpesviridae* viruses was recently proposed [31].

G4Hunter is one of several software programs available for predicting putative G-quadruplex forming sequences (PQS) [32,33,34]. The G4Hunter algorithm searches for Gs/Cs and sums up the scores for the groups of bases. The final score is thus a combination of G-richness and G-skewness and the presence of G-blocks. The default threshold is set to a G4Hunter score of 1.2, which has proven to be a reasonable compromise between false-negative and false-positive results. The higher the score, the higher the probability for a G4 structure to form [33]. G4Hunter provides the benefit of targeting even atypical G4s that could not be found by pattern-based algorithms [35,36]. 

Here, we present an extensive analysis of 2903 viruses across a diverse range of host organisms. Our goals were to identify PQS occurrence and localization in the genomes of viruses infecting a given host group, study the evolutionary differences related to PQS, and describe the potential dependence of PQS frequency between a virus and the corresponding host. 

## 2. Results and Discussion

### 2.1. Variation in Frequency for G4-Forming Sequences in dsDNA Viruses Grouped by Host

**Figure 1:**
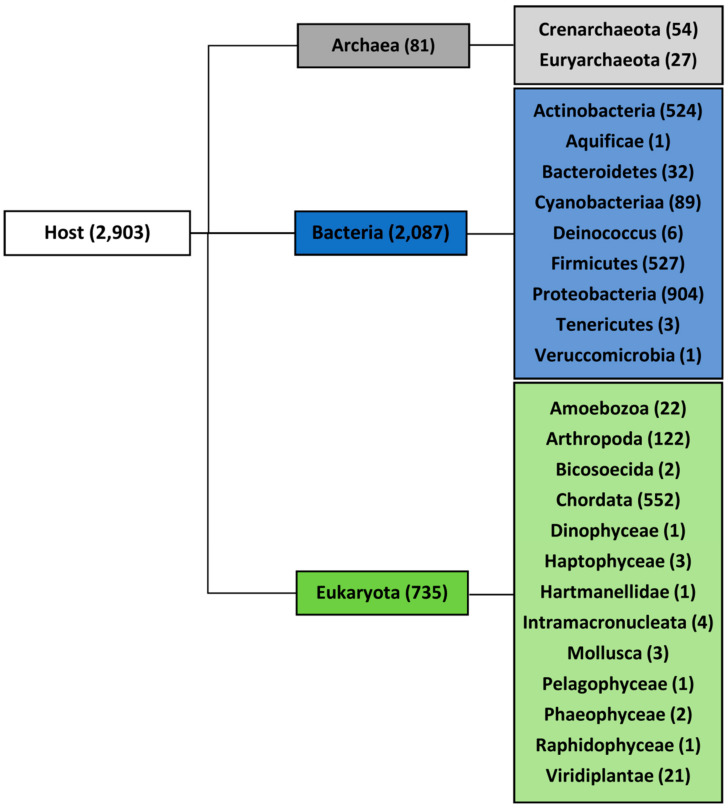
Full set of viral genomes divided according to host. The number of accessible unique genomes for each domain and group is shown in brackets.

Using the National Center for Biotechnology Information (NCBI) taxonomy classification, the analyzed viruses were divided into three domains according to their host organisms: Archaea, Bacteria, and Eukaryota. The domains were further divided into 23 groups (12 with five or more sequenced genomes) as shown in the phylogenetic tree in Figure 1. Phylogenetic classification of the viruses and corresponding host is presented in Supplementary Materials S1. All hosts were assigned by the Virus-Host database without further modification [37], which could have limited the potential host range, especially in arboviruses. Whereas 95% of all known bacteriophages and archaea-infecting viruses have dsDNA genomes [38], eukaryotes could be infected by all classes of the Baltimore virus classification, which means, in addition to dsDNA viruses, also ssDNA viruses, dsRNA viruses, ssRNA viruses, ssRNA reverse-transcribing viruses, and dsDNA reverse-transcribing viruses. We therefore restricted the analyses of PQS occurrence to only dsDNA viruses, although they have not been found to infect higher plants belonging to the Streptophyta but only lower species of plants belonging to Chlorophyta [39,40].

For further statistical analyses, only those groups with five or more sequenced genomes were included. We analyzed the PQS occurrence in all 2903 reference dsDNA viral genomes divided according to their host organisms (Supplementary Materials S2). A summary of all PQS found in ranges of the G4Hunter score intervals (1.2–1.4, 1.4–1.6, 1.6–1.8, 1.8–2.0, and 2.0 and higher) and precomputed PQS frequencies per 1000 nt is shown in Table 1. 

The mean frequency for all viral genomes in G4Hunter score interval 1.2–1.4 was 1.27 PQS per 1000 nt (see above). The lowest frequency in the same interval was observed for bacteriophages (0.88 PQS per 1000 nt), whereas the highest frequency was detected for archaea-infecting viruses (1.74). Surprisingly, there was not one PQS with a G4Hunter score higher than 1.4 found in the archaea host domain. In the Bacteria and Eukaryota host domains, by contrast, there were some PQS found even with G4Hunter scores higher than 2.0.

**Figure 2:**
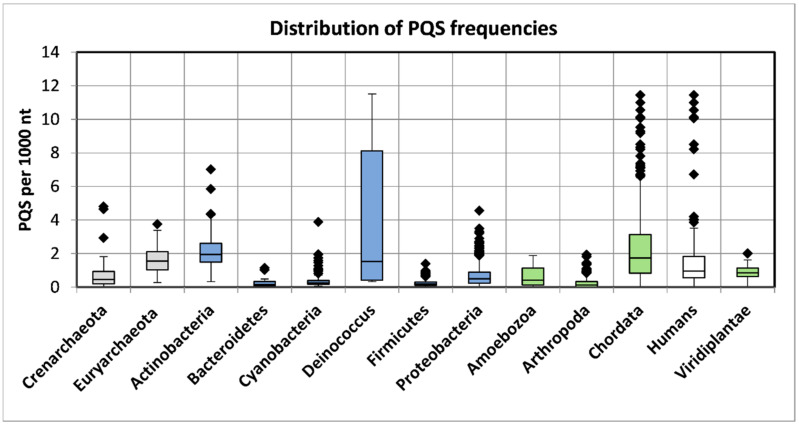
Frequencies of PQS in host groups of the analyzed viral genomes. Data within boxes span the interquartile range and whiskers show the lowest and highest values within the 1.5 interquartile range. Black diamonds denote outliers. The colors correspond to phylogenetic tree depiction.

**Figure 3:**
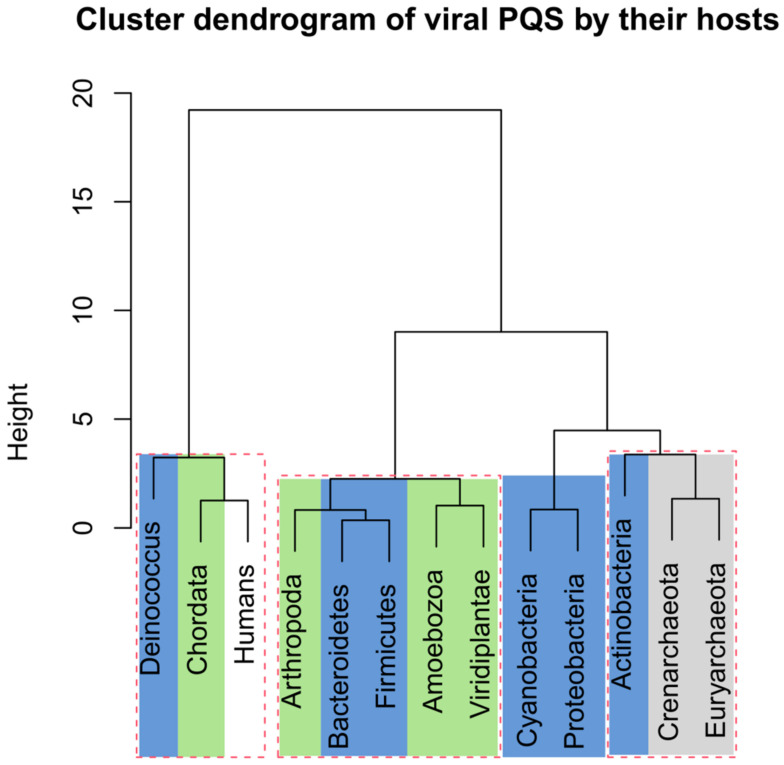
Cluster dendrogram based on PQS characteristics in all viral species by their host. Input data are listed in Supplementary Materials S4. Statistically significant clusters (based upon approximately unbiased *p*-values above 95, equivalent to *p*-values lower than 0.05) are highlighted by rectangles drawn with broken red lines. The colors correspond to phylogenetic tree depiction.

The numbers of analyzed viral sequences, grouped by their host phylogenetic categories; median genome length; mean, minimum, and maximum observed frequency of PQS per 1000 nt; and total PQS counts are summarized in Table 2. Just four viral groups (viruses infecting Euryarchaeota, Actinobacteria, *Deinococcus*, and Proteobacteria) showed >50% GC content. On the other hand, three groups (viruses infecting Bacteroidetes, Firmicutes, and Arthropoda) showed <40% GC content. Detailed statistical characteristics for PQS frequencies per 1000 nt (including mean, variance, and outliers) are depicted in boxplots for all inspected host groups in Figure 2. The mean frequency for all viral genomes was 1.32 PQS per 1000 nt. Detailed statistical analyses of host inter-domain and intergroup comparisons are presented in Supplementary Materials S3. We observed the highest mean frequency in the archaea host domain (1.76), followed by viruses infecting Eukaryota (1.52), whereas the lowest was noted in bacteriophages (0.89). At the group level, the most extreme values were found within the viruses infecting the bacteria domain. The lowest mean frequency was found in viruses infecting the Firmicutes (0.32) followed by Bacteroidetes (0.41). The highest PQS frequency was observed in the *Deinococcus* host group (4.21), followed by Actinobacteria (2.27). In viruses infecting the archaea domain, notable enrichment relative to the average was found for both the Euryarchaeota (1.69) and Crenarchaeota (1.85) groups. Within the Eukaryota host domain, the highest PQS frequency was observed for Chordata (2.18), the lowest for Arthropoda (0.30). The mean PQS frequency found in viruses infecting humans was lower (1.75) than the average PQS in the Chordata host group as normalized by the number of viruses infecting one host genus (2.18). We created a cluster dendrogram, as shown in Figure 3, to further reveal and graphically depict similarities among host groups. The input data and R code are listed in Supplementary Materials S4. Viruses infecting humans are notably clustered together with other viruses infecting Chordata on the left side of the dendrogram. Other viruses infecting eukaryotes are clustered in the second branch on the right. 

### 2.2. Features Characteristic for Hosts Are Enriched for PQS in Corresponding dsDNA Viral Genomes

**Figure 4:**
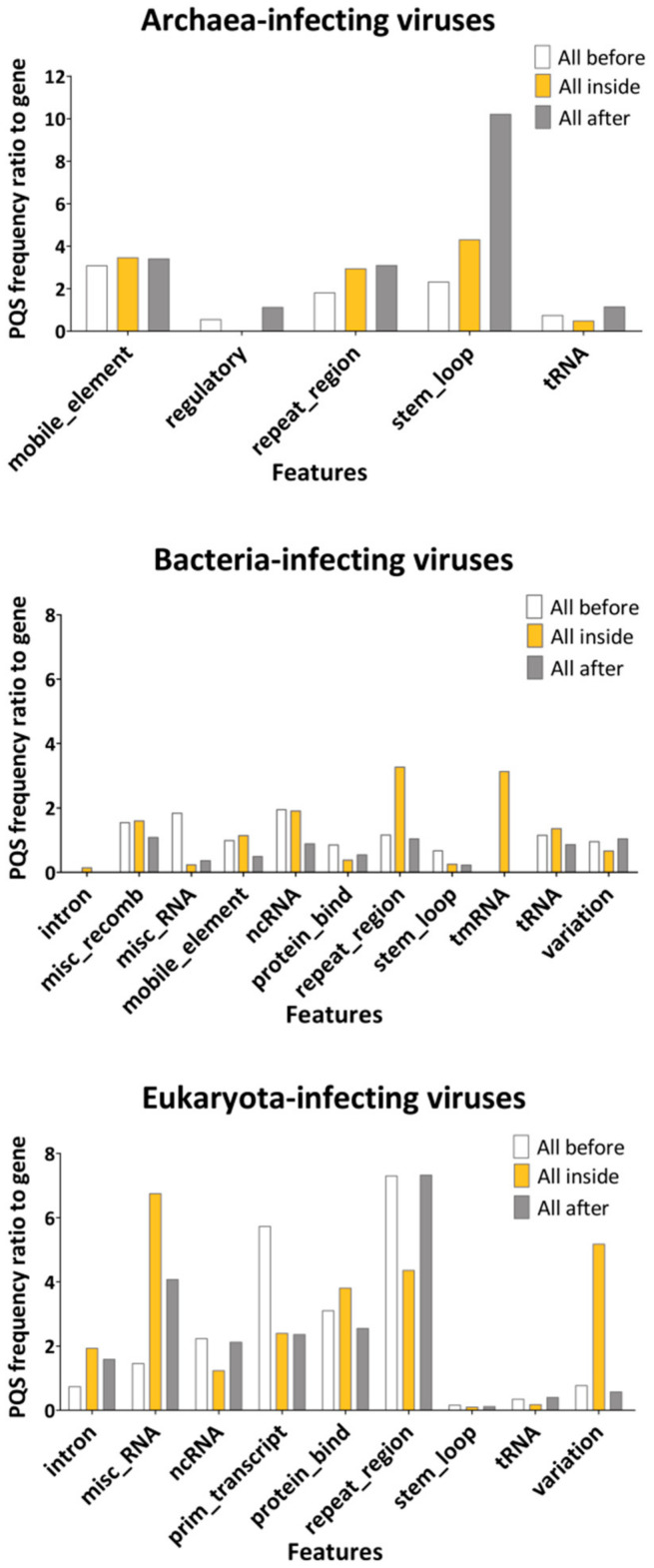
The ratio of PQS frequencies per 1000 nt between gene annotation and other annotated locations from the NCBI database. PQS frequencies within (inside), before (100 nt), and after (100 nt) annotated locations were analyzed. Detailed results are summarized in Supplementary Materials S5.

To evaluate the localization of PQS within viral genomes, we overlapped PQS with annotation regions extracted from the NCBI database (Supplementary Materials S5). We took the PQS frequency per 1000 nt in genes as a reference and plotted the ratio of the PQS frequency in features to that in genes (Figure 4). PQS frequencies differ depending on the annotated motif and across different hosts. In the archaea domain, the most notable enrichment was found inside and 100 bp after *stem_loops* (4.2× and 10.2× enrichment) and *mobile_elements* (3.5× and 3.4× enhancement). Predictably, abundance was also found in the archaea-infecting viruses’ *repeat_regions* (2.9×). The *repeat_regions* were also enriched for PQS inside bacteria-infecting (3.3×) and Eukaryota-infecting viruses (4.4×). The highest relative frequency inside bacteria was found in *tmRNA* (3.13) and *ncRNA* (1.9×), followed by a region 100 bp long before *misc_RNA* and *misc_recomb* (1.8 and 1.5× abundance). In addition to *repeat_regions*, we noted PQS enrichment in *misc_RNA* (6.7×), *variation* (5.2×)*, protein_bind* (3.8×), and *introns* (1.9×) of Eukaryota-infecting viruses. Notably, the PQS frequency was increased in comparison to *genes* inside *introns* only in Eukaryota-infecting viruses (1.9× enrichment), whereas *introns* in bacteria-infecting viruses were depleted for PQS presence (0.14× lower PQS frequency in comparison to genes). This indicates that the prevalence of PQS in specific viral features is important for the host’s cellular machinery. A G4 located in an intron could affect the expression profile; it was shown to regulate the splicing of alternative isoforms of a p53 protein in the human genome [41].

### 2.3. PQS Frequencies of dsDNA Viruses Correlate with Their Hosts’ Genomes

**Figure 5:**
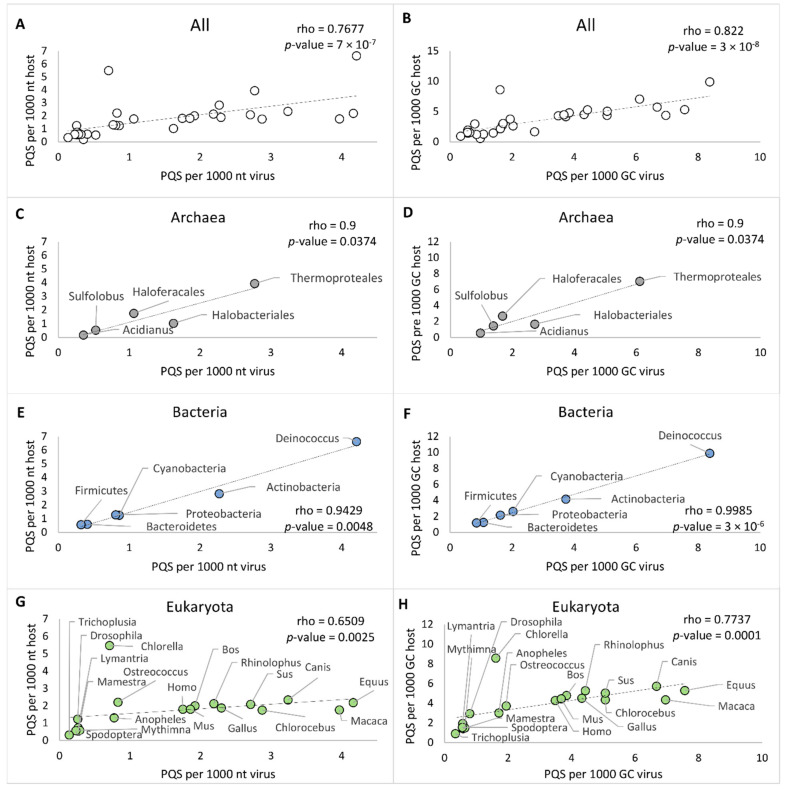
Relationships between virus and various hosts as measured by observed PQS frequency per 1000 nt and PQS frequency per 1000 GC. (**A**) All host-virus pairs, PQS frequencies; (**B**) All host–virus pairs, PQS per 1000 GC; (**C**) Archaea–virus pairs, PQS frequencies; (**D**) Archaea–virus pairs, PQS per 1000 GC; (**E**) Bacteria–virus pairs, PQS frequencies; (**F**) Bacteria–virus pairs, PQS per 1000 GC; (**G**) Eukaryota–virus pairs, PQS frequencies; (**H**) Eukaryota–virus pairs, PQS per 1000 GC of the archaea–virus pairs.

Next, to evaluate the relationship between PQS frequencies of the virus and the host, we analyzed selected genomes of host organisms for PQS presence. For hosts from archaea and bacteria, we utilized the previously published results of our group on PQS occurrence in all accessible archaeal and bacterial genomes [19,42]. In all analyses, we used the same workflow and same parameters for G4Hunter and data processing. Reference genomes of the Eukaryota hosts were retrieved from the NCBI database, and their list together with correlation analyses is available in Supplementary Materials S1. We selected all available reference genomes of hosts belonging to Viridiplantae, and for the remaining Eukaryota groups (Arthropoda and Chordata), we selected the 10 most frequent hosts in each corresponding category. There is no reference genome, however, for the *Acanthamoeba* genus, the only host of Amoebozoa-infecting viruses. We always compared a single eukaryotic host genome to all corresponding dsDNA viruses. The overall results of the correlation analyses are presented in Figure 5 and in the Supplementary Material S6. Spearman’s correlation coefficient for the average of PQS frequencies in all investigated virus-host pairs was determined to be 0.7677, with a statistically significant *p*-value of 7 × 10^−7^ (Figure 5A). To exclude the GC content as a bias factor, we plotted also the average PQS/GC per 1000 nt. The correlation coefficient for the average of PQS/GC per 1000 nt then increased to 0.822, with *p*-value of 3 × 10^−8^ (Figure 5B).

The strongest correlation was found between virus–bacteria pairs. Spearman’s correlation coefficient for PQS frequency of bacteria-infecting viruses and their hosts showed a strong, statistically significant (*p*-value < 0.01) positive correlation (Figure 5E,F). The correlation coefficient was 0.9429 for PQS frequency and 0.9985 for GC/PQS, with *p*-value < 0.01. Our previously published PQS frequencies of all known bacterial genomes [19] correspond to the frequencies determined here for PQS in bacteriophage genomes. In all virus–bacteria pairs, the mean PQS frequency was higher in the bacterial group than in the viral host group. A statistically significant positive correlation (*p*-value < 0.05) was observed, also with PQS frequencies grouped by G4Hunter score intervals and PQS frequencies identified by the Tetraplex Finder module of QuadBase2 software with default low stringency parameters (Supplementary Materials S7). Dispersion of PQS frequencies among bacteriophages was more diverse than inside other viruses, and the same observation (higher diversity in PQS frequencies) has been reported for bacteria compared to other hosts [18]. The corresponding frequencies of virus and bacteria hosts confirmed by correlation analyses pointed to their having close coevolutionary processes. The second highest correlation coefficient was found for the Archaea subgroup, with a value of 0.9 for PQS average frequency and PQS/GC (*p*-value < 0.05) (Figure 5C,D). To distinguish several different phyla, we further divided Crenarchaeota into two subgroups (*Sulfolobus*, Thermoproteales) and Euryarchaeota into three subgroups (*Acidianus*, Halobacteriales, Haloferacales). Because of the high diversity and the low number of sequenced genomes in several categories, the minimum number of viral or host genomes for statistical analyses was set to four.

Inside the eukaryote domain, we noted lower but still statistically significant (*p*-value < 0.01) correlation coefficients of 0.6509 for PQS frequencies and 0.7737 for PQS/GC. This finding could be attributed to two main causes (Figure 5G,H). First, the statistical sample for Eukaryota host genomes was significantly smaller, in comparison to that for bacteria and archaea host domains; an average of six host genomes were analyzed for each group of Eukaryota, shown in Table 2. Second, with the increasing complexity of the organisms, genomic duplications, and extensive repetitions, the correlation could be less obvious and significant.

Recently, coevolution of G4 sequence composition between dsDNA viruses from the *Herpesviridae* family and host has been proposed, as herpesviruses are often enriched for C-rich looped G4s, which are binding sites for host transcription factors, and TTA-looped G4s identical to the telomeric motif of vertebrates [31]. Mimicking the genome organization of the host could influence the PQS prevalence in dsDNA viral genomes and vice versa. 

## 3. Materials and Methods 

### 3.1. Viral and Host Sequences

A total of 3134 sequences of 2903 unique viral genomes were downloaded from the genome database of the National Center for Biotechnology Information (NCBI). Where more than one sequence was available, only the reference genome was used in the analyses. Hosts were assigned according to the NCBI and the Virus-Host database [37]. Subviral agents were assigned to the host of the coinfected virus as stated in the database [37]. A full list of NCBI IDs and host assignments are presented in Supplementary Materials S1. For hosts from archaea and bacteria, we utilized the previously published results of our group on PQS occurrence in all accessible archaeal and bacterial genomes [19,42]. For eukaryote groups, we selected the 10 most frequented hosts for each viral group, and these are also listed in Supplementary Materials S1.

### 3.2. PQS Analyses

Analyses were run using the computational core of DNA analyzer software written in Java [43] with G4Hunter algorithm implementation [32] and default parameters (25 nt for window size, G4 score threshold 1.2). The overall results as to the number of PQS found together with the size of genomic DNA, GC content, PQS frequency normalized for 1000 nt, and lengths of sequences covered with PQS are summarized in Supplementary Materials S2. The average PQS frequency of host groups, shown in Table 1, was normalized by the number of viruses infecting each genus to avoid sampling bias due to the overabundance of viruses infecting specific species (such as a human). PQS were also classified into the five intervals of the G4Hunter score: 1.2–1.4, 1.4–1.6, 1.6–1.8, 1.8–2.0, and 2.0 and more. To confirm the results acquired by G4Hunter, bacteriophages and corresponding hosts were selected and analyzed by the Tetraplex Finder module of QuadBase2 software with default low stringency parameters (nonoverlapping PQS with minimum two-tracked PQS and loop length of 1–12 nt), and the results are listed in Supplementary Materials S7 [44].

### 3.3. Statistical Analysis

The normality of data was tested by the Shapiro–Wilk test. The nonparametric Kruskal–Wallis test was utilized for statistical evaluations of the differences in PQS among host phylogenetic groups. Post hoc multiple pairwise comparisons, using Dunn’s test with Bonferroni correction of the significance level, were applied with the *p*-value cutoff set at 0.05; data are available in Supplementary Materials S3 for sequences grouped by their host organism and their comparison to the PQS frequency of host groups. For correlation analyses, the two-tailed Spearman’s correlation coefficient was considered. To further reveal and graphically depict similarities between viral hosts, we constructed a cluster dendrogram of PQS characteristics in program R, version 3.6.3, using the pvclust package. The following values were used as input data: Mean f (mean of predicted PQS per 1000 nt), Min f (the lowest frequency of predicted PQS per 1000 nt), Max f (the highest frequency of predicted PQS per 1000 nt), and Cov % (% of genome covered by PQS) (Supplementary Materials S4). The following parameters were used for both analyses, the cluster method “ward.D2”, distance “euclidean”, and the number of bootstrap resampling 10,000. Statistically significant clusters (based on approximately unbiased *p*-values values above 95, equivalent to *p*-values less than 0.05) are highlighted in Figure 3 by rectangles marked with broken red lines. R code is provided in Supplementary Materials S4.

### 3.4. Overlay of PQS with Annotated Features from NCBI 

Annotated feature tables of all viral genomes were downloaded from the NCBI database. Features were grouped by their names as stated in the feature table file. PQS occurrence was analyzed inside and around (before and after) a predefined featured neighborhood (±100 nt). From this analysis, we obtained a file with feature names and numbers of PQS found inside and around features. Further processing was performed in Microsoft Excel and the data are available as Supplementary Material S5.

## 4. Conclusions

PQS frequencies in viral genomes differ across host taxa and correspond to the PQS frequencies of the host organism. The overlay of PQS with annotated regions revealed nonrandom localization of G4 sequences and their abundance in various regions, such as repeat regions, stem-loops, mobile elements, protein-binding regions, RNA, etc. While abundance and depletion in some locations are shared by viruses of different hosts, others are unique. For example, there is an abundance of PQS in introns of Eukaryota-infecting viruses, but depletion of PQS in introns of bacteria-infecting viruses. Our study revealed the correlation between PQS frequencies of dsDNA viruses and corresponding hosts from archaea, bacteria, and even eukaryotes, which indicate their close coevolution and evolutionarily reciprocal mimicking of genome organization.

## Figures and Tables

**Table 1:** Total number of putative G-quadruplex-forming sequences (PQS) and their resulting frequencies per 1000 nt in all 2903 viral genomes and host categories, grouped by G4Hunter score. Frequencies were calculated as the total number of PQS in each category divided by the total length of all analyzed sequences, multiplied by 1000 and normalized by the number of viruses infecting one genus.

G4Hunter Score	PQS Frequency per 1000 nt			
	All	Archaea	Bacteria	Eukaryota
1.2–1.4	1.27	1.74	0.88	1.46
1.4–1.6	0.039	0.025	0.026	0.047
1.6–1.8	0.0042	0	0.00088	0.0062
1.8–2.0	0.00025	0	0.000041	0.00038
2.0 and more	0.00021	0	0.000050	0.00031

**Table 2:** Distribution of PQS frequencies in viruses according to host organisms. Genomic length, PQS frequencies, and total counts. Seq (total number of sequences), Median (median length of sequences), GC% (average GC content), PQS (total number of predicted PQS), Mean f (mean frequency of predicted PQS per 1000 nt normalized by the number of viruses infecting one genus), Min f (the lowest frequency of predicted PQS per 1000 nt), Max f (the highest frequency of predicted PQS per 1000 nt), and Cov (% of genome covered by PQS).

**All**	**Seq**	**Median**	**GC%**	**PQS**	**Mean f**	**Min f**	**Max f**	**Cov **
All	3134	44,746.5	44.94	220,569	1.32	0	11.51	3.34
**Domain**	**Seq**	**Median**	**GC%**	**PQS **	**Mean f**	**Min f**	**Max f**	**Cov **
Archaea	81	33,356	48.92	3137	1.76	0	4.80	4.32
Bacteria	2087	49,639	48.10	112,664	0.89	0	11.51	2.11
Eukaryota	966	7951.5	43.09	104,768	1.52	0	11.44	3.93
**Group**	**Seq**	**Median**	**GC%**	**PQS **	**Mean f**	**Min f**	**Max f**	**Cov **
Crenarchaeota	54	32,047.5	40.91	1012	1.85	0	4.80	4.76
Euryarchaeota	27	49,107	54.92	2125	1.69	0.28	3.75	3.99
Actinobacteria	524	53,403.5	60.90	61,313	2.27	0.33	7.02	5.12
Bacteroidetes	32	47,060	38.12	477	0.41	0.03	1.14	1.01
Cyanobacteria	89	174,079	43.33	3875	0.82	0.06	3.88	2.10
*Deinococcus*	6	61,150	50.26	726	4.21	0.33	11.51	10.45
Firmicutes	527	41,843	38.14	7886	0.32	0	1.39	0.78
Proteobacteria	904	49,035	50.07	38,334	0.80	0	4.55	1.90
Amoebozoa	22	495,022	42.47	21,931	0.66	0	1.89	1.60
Arthropoda	345	7276	38.77	4957	0.30	0	1.92	0.73
Chordata	561	7852	45.48	72,420	2.18	0	11.44	5.65
Viridiplantae	21	193,301	46.91	3542	1.06	0	2.01	2.54
**Subgroup**	**Seq**	**Median**	**GC%**	**PQS**	**Mean f**	**Min f**	**Max f**	**Cov **
Humans	120	7344	42.55	15,996	1.75	0	11.44	4.48

The colors correspond to phylogenetic tree depiction in Figure 1 (Grey—Archaea, Blue—Bacteria, Green—Eukaryota as host organisms).

## Data Availability

Not applicable.

## Supplementary Materials

Supplementary Materials can be found at https://www.mdpi.com/article/10.3390/ijms22073433/s1.

## Abbreviations

EBV	Epstein–Barr virus
G4s	G-quadruplexes
HBV	hepatitis B virus
HCV	hepatitis C virus
HIV	human immunodeficiency virus
KSHV	Kaposi sarcoma herpes virus
PQS	putative G-quadruplex-forming sequences
NCBI	National Center for Biotechnology Information
